# Severe dilated cardiomyopathy as an unusual clinical presentation in an infant with sialidosis type II

**DOI:** 10.1002/jmd2.12357

**Published:** 2023-01-07

**Authors:** Margot Eyskens, Luc Bruyndonckx, André B. P. Van Kuilenburg, François Eyskens

**Affiliations:** ^1^ Department of Paediatrics University of Antwerp Antwerp Belgium; ^2^ Cardiology, Department of Paediatrics University Hospital Antwerp Antwerp Belgium; ^3^ Laboratory Genetic Metabolic Diseases, Gastroenterology and Metabolism, Department of Clinical Chemistry Amsterdam University Medical Centers Amsterdam The Netherlands; ^4^ Inherited Metabolic Diseases, Department of Paediatrics University Hospital Antwerp Antwerp Belgium

**Keywords:** dilated cardiomyopathy, lysosomal storage disease, *NEU1* gene, neuraminidase, oligosaccharidosis, sialidosis type II

## Abstract

We report a unique case of an infant with a severe dilated cardiomyopathy as the clinical presentation of sialidosis type II (OMIM 256550), a rare autosomal recessive inherited lysosomal storage disease that is characterized by partial or complete deficiency of α‐neuraminidase, following mutations in the gene neuraminidase 1 (*NEU1*), located on the short arm of chromosome 6 (6p21.3). Accumulation of metabolic intermediates leads to severe morbidity, especially myoclonus, gait disturbances, cherry‐red macules with secondary loss of visual acuity, impaired color vision and night blindness, and sometimes additional neurological findings such as seizures. Dilated cardiomyopathies are characterized by dilation and impaired contraction of the left or both ventricles, whereas most of the metabolic cardiomyopathies are hypertrophic forms appearing with diastolic dysfunction and, in case of lysosomal storage diseases, often associated with valvular thickening and prolapse. Cardiac manifestations in systemic storage disorders are common although rarely described in mucolipidoses. In mucolipidosis type 2 or I‐cell disease only three cases were presented with severe dilated cardiomyopathy and endocardial fibroelastosis in infancy, as opposed to sialidosis type II, by which to the best of our knowledge no presentation of dilated cardiomyopathy was previously reported in literature.

**Synopsis**

In order to get prognostic information, we advise to perform detailed cardiac examinations as essential components of the diagnostic work‐up in patients with sialidosis type II.

## 1 INTRODUCTION

Sialidosis type II, is a lysosomal storage disorder, inherited as an autosomal recessive trait and characterized by a deficiency of the enzyme α‐neuraminidase, resulting in the abnormal accumulation of toxic materials in the body. Sialidosis type I usually becomes apparent during the second decade of life with the development of myoclonus, gait disturbances, nystagmus, opacity of the cornea, cherry‐red macules with secondary loss of visual acuity, impaired color vision and night blindness, and sometimes additional neurological findings such as seizures, hyperreflexia and ataxia, as opposed to sialidosis type II, which is usually more severe and often presents at birth or starts during infancy or early childhood as a genocopy of GM1‐gangliosidosis. Sialidosis type II is caused by mutations of the neuraminidase 1 (*NEU1*) gene, located on the short arm of chromosome 6 (6p21.3), resulting in a progressive lysosomal storage from sialylated glycoconjugates and oligosaccharides. It affects males and females in equal numbers. Specific treatments (enzyme replacement therapy) have not yet been developed whereas procedures for prenatal diagnosis are available and genetic counseling can be offered in this lysosomal storage disorder. Other treatment is symptomatic and supportive.^1^, ^2^

This case report will describe an infant with severe dilated cardiomyopathy as a clinical presentation of sialidosis type II (OMIM 256550).^3^

## 2 CASE PRESENTATION

A girl of Turkish origin was born at a postmenstrual age of 37 weeks and 2 days, after an uncomplicated pregnancy by an urgent secondary cesarean section because of fetal distress. She had an Apgar score of 8 at 1 min after birth, 9 at 5 min and 10 at 10 min and a normal birth weight of 3415 g (75th–90th percentile). Her length at birth was at the 90th percentile and her head circumference at the 50th–75th percentile. The TORCH panel test was negative. She was born to consanguineous parents who were second degree relatives, namely cousin and niece. She was the second child for these parents, with a healthy older sister. In the family history, the maternal grandmother had heart problems and hypertension, while two brothers of the father died at the ages of 3 months and 2 years, and a niece had 2 children who died as an infant or toddler.

From the neonatal period, she presented with failure to thrive, hypotonia, hepatomegaly, splenomegaly, ascites, dyspnea and coarse facial features, namely frontal bossing, a depressed nasal bridge and broad nasal tip, large low‐set ears, a long philtrum, gingival hypertrophy and macroglossia. Echocardiography revealed a dilated cardiomyopathy with poor myocardial contractility and extremely systolic and diastolic dysfunction of the left ventricle. There were no clinical, biochemical or echocardiographic signs of myocarditis. At the age of 3 weeks clinical signs of cardiac insufficiency worsened and a therapy with dobutamine and hydrochlorothiazide was implemented. At that time, there were no signs of cardiac decompensation. Three days later the medical treatment was changed to the association of enalapril, hydrochlorothiazide and spironolactone. Blood pressures remained stable. Cardiac function was weekly followed by echocardiography. Because of episodes of tachypnea, she was supported with a low flow nasal cannula with a maximum FiO_2_ of 0.25 during her stay at the neonatal intensive care. Transfontanellar ultrasound showed normal findings. She was periodically restless. The hearing test was bilateral disturbed (no details available). Ophthalmological examination was normal. During the neonatal period, she suffered from a pyelonephritis with a *K. pneumoniae*, and was treated with amikacin and vancomycin for 10 days. A slow biochemical and clinic response was seen. Parenteral feeding was given until the age of 3 weeks. Enteral feeding was then introduced and slowly augmented. At the age of 4 weeks, she was fully fed by nasogastric tube. Because of the findings of hepatomegaly and ascites, congenital infections were excluded, as well as a portal vein thrombosis. Hematological blood tests showed vacuolated lymphocytes, typical for a lysosomal storage disease. Ultrasound of the kidneys showed enlarged hyperechoic kidneys.

**FIGURE 1:**
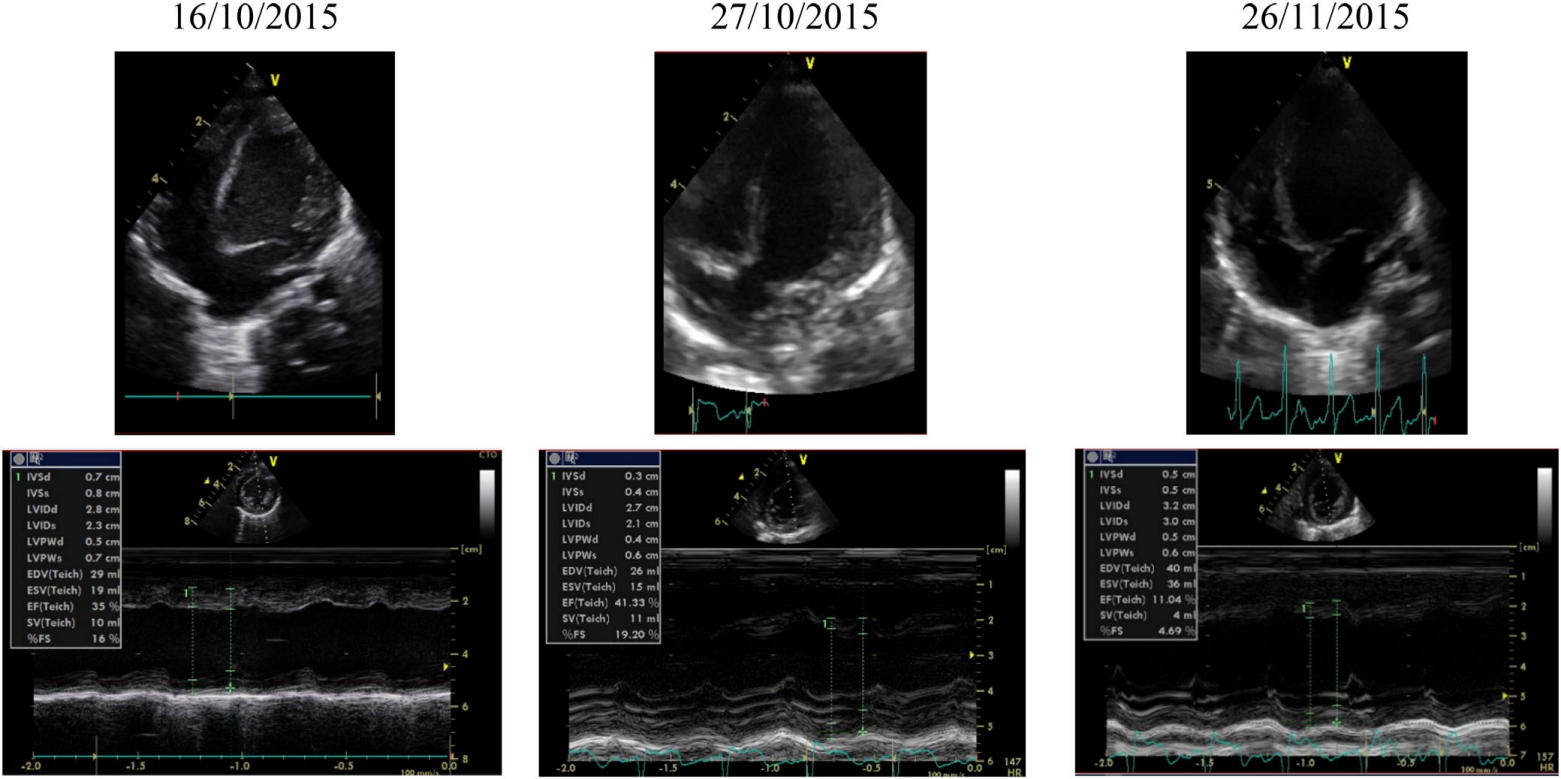
Serial echocardiographic findings during follow‐up of the described patient. Cardiac decompensation, secondary to the dilated cardiomyopathy, was rapidly progressing. Fractional shortening varied between 16% and 20% at the age of 2 months and between 4% and 11% at the age of 3 months.

Cardiac decompensation, secondary to the dilated cardiomyopathy, was rapidly progressing and followed by several hospitalizations, despite treatment with oxygen therapy, diuretics, an ACE‐inhibitor associated with the beta blocker carvedilol and feeding by a nasogastric tube. ECG showed large P waves and a hypertrophy of the left ventricle. Fractional shortening varied between 16% and 20% at the age of 2 months and between 4% and 11% at the age of 3 months. Left ventricle end diastolic diameter was 32 mm and there were high filling pressures with retrograde pulmonary hypertension (Figure 1).

Palliative care was initiated. She died at the age of 11 months of cardiac decompensation and arrest.

**FIGURE 2:**
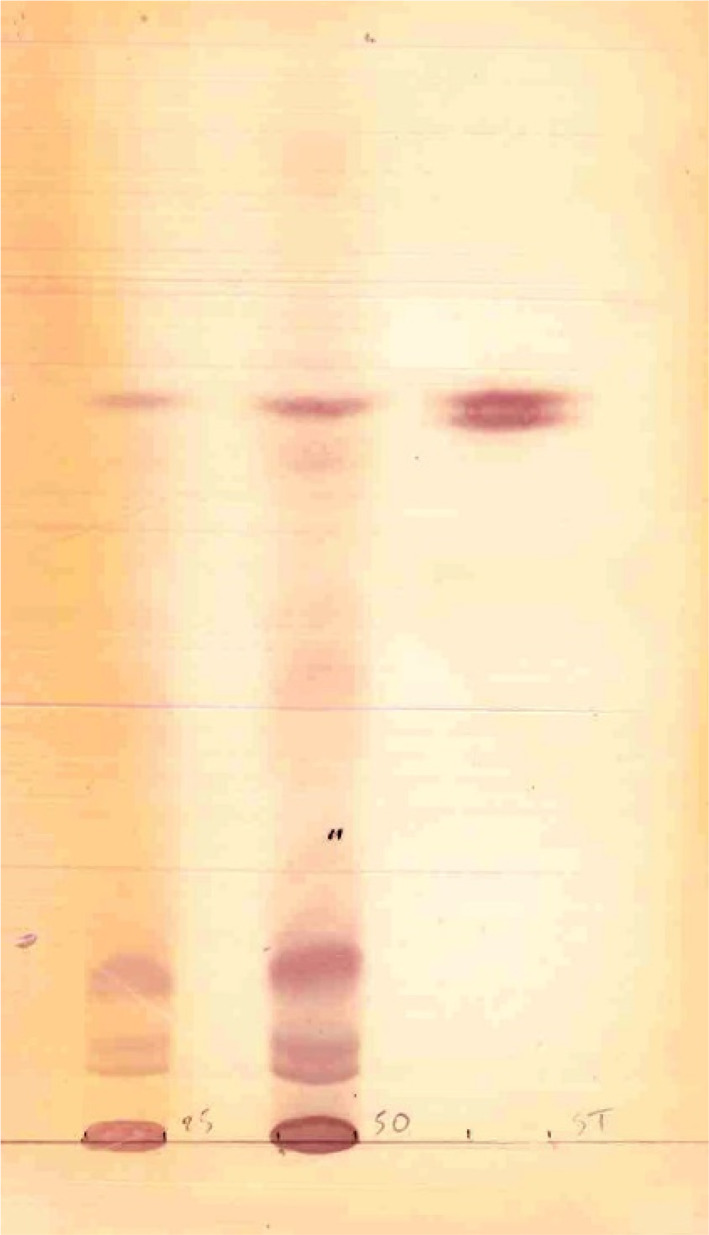
Oligosaccharides in urine analyzed by thin layer chromatography. Lane 1 shows the result of the patient. This pattern is suggestive of sialidosis type II. In comparison, lane 2 shows the result of a patient diagnosed with infantile GM1 gangliosidosis.

After extensive biochemical metabolic testing, an inherited lysosomal storage disorder was assumed, in particular a GM1 gangliosidosis. There was a great urinary excretion of sialic acid. Analysis of oligosaccharides^4^ was compatible with a galactosialidosis, due to protective protein/cathepsin‐A deficiency, causing a combined deficiency of neuraminidase and beta‐galactosidase (Figure 2). The differential diagnosis with sialidosis type II was made. Enzymatic analysis of cultured fibroblasts (analysis performed at Amsterdam University Medical Centers^5^) showed a severe deficient activity of N‐acetyl‐α‐d‐neuraminidase (0.3 nmol/mg h (RW 15.0–45.0)) (Table 1), in combination with a normal or slightly raised activity of beta‐d‐galactosidase and protective protein/cathepsin A, compatible with the diagnosis of sialidosis type II and excluding galactosialidosis.

**TABLE 1:** Enzyme analysis of cultured fibroblasts showed a severe deficient activity of N‐acetyl‐α‐d‐neuraminidase, in combination with a normal or slightly raised activity of beta‐galactosidase and protective protein/cathepsin A, compatible with the diagnosis of sialidosis type II and excluding galactosialidosis.

	Patient	Reference values	SI units
N‐acetyl‐α‐d‐neuraminidase	0.3	15.0–45.0	nmol/mg h
Protective protein/cathepsin A	6926	2500–5500	nmol/mg h
Beta‐d‐galactosidase	1215	600–1650	nmol/mg h

Genetic testing (University Hospital Brussels; method:capture‐based panel, using the NEBNext Ultra II FS DNA Library Prep Kit for Illumina and IDT xGen Exome Hybridization Panel (64 genes). PCR amplification and DNA sequencing of the coding regions of the 6 exons of the *NEU1* genewith self‐designed primers (sequencies are available on request), as well as apart of their flanking introns. This method detects 〉 95% of the mutations in this gene. Large genomic deletions and duplications are not always detected.) resulted in a molecular confirmation of α‐neuraminidase deficiency. She was homozygous for c.625delG, p.Glu209Serfs*94 mutation in exon 4 of the *NEU1* gene. This results in a frameshift and a premature stop codon. The possibility of preimplantation genetic testing was proposed to the parents for future pregnancies.

## 3 DISCUSSION

Dilated cardiomyopathies are the most common type of cardiomyopathy in early childhood. Approximately 30% are genetic cardiomyopathies of autosomal dominant, X‐linked or mitochondrial inheritance pattern. In about 20%, sequels of myocarditis or endocardial fibroelastosis, anomalies of coronary arteries or previous therapy with doxorubicin lead to secondary dilated cardiomyopathies and about 50% of the dilated cardiomyopathies have to be classified as idiopathic variants.^6^, ^7^

Dilated cardiomyopathies are characterized by dilation and impaired contraction of the left or both ventricles, whereas most of the metabolic cardiomyopathies are hypertrophic forms appearing with diastolic dysfunction and, in case of lysosomal storage diseases, often associated with valvular thickening and prolapse.^7^ Cardiac manifestations in systemic storage disorders are common although rarely described in mucolipidoses.^8^ In mucolipidosis type 2 there are a few reports of involvement of the cardiovascular system during the first weeks of life: three cases were presented with severe dilated cardiomyopathy and endocardial fibroelastosis in infancy.^7^, ^9^, ^10^

To our knowledge, we report the first case of an infant with severe dilated cardiomyopathy affected by sialidosis type II. Other inherited metabolic disorders, such as fatty acid oxidation disorders and mitochondrial diseases, as possible underlying diseases, were excluded.^6^ The clinical presentation in this patient differs from the cardiac manifestations in patients with the early‐onset severe form of galactosialidosis, in which cardiomegaly, a thickened septum, and secondary cardiac failure are often described. Patients with galactosialidosis die at an average age of 7 months, probably as a result of renal and cardiac failure.^11^

Pathophysiologic mechanisms of the cardiac involvement described in this patient are not clear. In order to get prognostic information we advise to perform detailed cardiac examinations as essential components of the diagnostic work‐up in patients with sialidosis type II.^7^

## 4 CONCLUSION

We report a first case of an infant with severe dilated cardiomyopathy affected by the lysosomal storage disease sialidosis type II.

## AUTHOR CONTRIBUTIONS

Margot Eyskens was responsible for the data collection and formulation of the manuscript. François Eyskens was the attending physician, responsible for the treatment plan and counseling during the disease course of this patient. Luc Bruyndonckx reviewed the cardiac information in this manuscript, André B. P. Van Kuilenburg provided information on the method of the enzymatic analysis of cultured fibroblasts and François Eyskens did the primary review before submission for external review. All authors approved the final version of the manuscript before submission.

## FUNDING INFORMATION

This research received no specific grant from any funding agency in the public, commercial, or not‐for‐profit sectors.

## CONFLICT OF INTEREST

The authors declare that they have no conflict of interest.

## INFORMED CONSENT

This article does not contain any studies with human or animal subjects performed by any of the authors.

## Data Availability

This manuscript has associated data in a data repository, including all data for which data deposition is mandatory.
